# Colon inflammatory index as a useful prognostic marker after R0 resection in patients with colorectal cancer liver metastasis

**DOI:** 10.1371/journal.pone.0273167

**Published:** 2022-10-04

**Authors:** Mitsutoshi Ishii, Tetsuro Tominaga, Takashi Nonaka, Shosaburo Oyama, Masaaki Moriyama, Keizaburo Maruyama, Terumitsu Sawai, Takeshi Nagayasu

**Affiliations:** 1 Department of Surgical Oncology, Nagasaki University Graduate School of Biomedical Science, Nagasaki, Japan; 2 Department of Surgery, Isahaya General Hospital, Isahaya, Nagasaki, Japan; Stanford University School of Medicine, UNITED STATES

## Abstract

**Background:**

Although R0 resection for colorectal cancer liver metastasis (CRLM) is a promising treatment with improved prognosis, the recurrence rate is still high. No prognostic markers have been reported after resection of CRLM. In this study, we investigated the association between inflammation-based score and prognosis after R0 resection in patients with CRLM.

**Methods:**

We retrospectively investigated 90 patients who underwent R0 resection for CRLM between 2008 and 2018. We calculated colon inflammatory index (CII) (CII0, low risk; CII1, intermediate risk; and CII2, high risk), modified Glasgow prognostic score, prognostic nutritional index, and CRP-to-albumin ratio; and retrospectively assessed the relationship between these scores, the clinicopathological features, and prognosis.

**Results:**

The median follow-up period was 44 months (range, 2–101 months). Five-year relapse-free survival (RFS) (CII2; 12.5%, CII1; 14.5%, CII0; 42.9%) and 5-year overall survival (OS) (CII2; 32.4%, CII1; 25.4%, CII0; 57.7%) were significantly lower in the high CII groups (CII1–2) compared with the low CII group (CII0) (p = 0.021 and p = 0.006, respectively). CEA level was significantly higher in the high CII group than the low CII group (12.4 vs 7.3, p = 0.004). Multivariate analysis showed CII score as an independent predictor of RFS (hazard ratio 2.128, 95%CI 1.147–3.430, p = 0.015) and OS (hazard ratio 2.639, 95%CI 1.331–5.323, p = 0.005).

**Conclusion:**

CII shows promise as a prognostic marker after R0 liver resection in patients with CRLM.

## Introduction

Colorectal cancer (CRC) is the second leading cause of cancer death in the world and has been on the rise in recent years [1]. Twenty percent of patients with CRC have distant metastases at presentation, and their overall 5-year survival is less than 20% [2]. R0 resection for colorectal liver metastasis (CRLM) is a promising treatment with improved prognosis, with a reported 5-year survival rate of approximately 25% (range, 4%–60%) [3]. However, the postoperative recurrence rate is reported to be as high as 70%–80%, and few prognostic scores have been reported [4, 5].

Inflammation-based score (IBS) has recently attracted attention as a predictor of prognosis in various tumors [6–8]. Activation of inflammation by tumors may be associated with inhibition of apoptosis and promotion of angiogenesis. Circulating lymphocytes exert antitumor activity by inducing cytotoxic cell death and inhibiting tumor growth. Colon inflammatory index (CII), which is a novel IBS, scores inflammation using neutrophil, lymphocyte, and lactate dehydrogenase (LDH) levels and has shown potential prognostic value in predicting CRC [9]. To the best of our knowledge, no study has reported an association between CII and CRLM.

The aim of the present study was to investigate the relationship between CII and various types of IBS and prognosis after R0 resection of CRLM.

## Materials and methods

We retrospectively investigated patients who had undergone R0 resection for CRLM at Nagasaki University Hospital between 2008 and 2018. Patients with incomplete laboratory data and who received emergency surgery were excluded. A final total of 90 patients were eligible for this analysis. This research study was conducted retrospectively from data obtained for clinical purposes, and was reviewed and approved by the Clinical Research Review Board of Nagasaki University Hospital (approval No. 16062715–5). and was conducted according to the principles of the Declaration of Helsinki. Written informed consent was obtained from the patient for publication of this study.

The types of IBS investigated in this study were CII, modified Glasgow Prognostic Score (mGPS), prognostic nutrition index (PNI), neutrophil-to-lymphocyte ratio (NLR), and CRP-to-albumin ratio (CAR).

CII combines the NLR with LDH. NLR is expressed as the serum neutrophil count (mg/dL) divided by the serum lymphocyte count (g/dL). Patients were divided into three risk groups according to CII value: CII0, low risk (0 factors: NLR <3 and LDH ≤upper limit of normal [ULN]); CII1, intermediate risk (1 factor: NLR ≥3 or LDH > ULN); and CII2, high risk (2 factors: NLR ≥3 and LDH > ULN). In the analysis, patients with CII scores 1–2 were defined as the high-risk group for analysis due to the small number of patients with a CII score of 2. The cutoff of NLR ≥3 was defined according to Passardi A et al. and the ULN for LDH was defined according to the limits used at our institution [10].

mGPS is based on serum levels of C-reactive protein (CRP) and albumin and is scored from 0 to 2. It was calculated as follows: score of 0, CRP ≤1.0 mg/dL; score of 1, CRP > 1.0 mg/dL; score of 2, CRP>1.0 mg/dL and albumin <3.5 g/dL [11].

PNI was calculated as 10 × the serum albumin (g/dL) + 0.005 × total lymphocyte count (/μL).

CAR was calculated by dividing serum CRP (mg/dL) by serum albumin (g/dL), and the cut-off values were set using ROC for continuous data.

Patients were divided into two groups for each IBS as follows: CII (0 vs 1–2), mGPS (0 vs 1–2), PNI (>49.3 vs ≤49.3), and CAR (>0.055 vs ≤0.055); and 5-year-relapse-free survival (RFS) and 5-year-overall survival (OS) were compared between the groups. In addition, clinicopathological characteristics were compared in two groups: high CII group (CII1–2, n = 33) and low CII group (CII0, n = 57).

The following clinical data were collected and compared between the CII high and CII low groups: sex, age at surgery, location of primary tumor, pathological T status, pathological N status, type of liver metastasis, surgical procedure, number of liver metastases, carcinoembryonic antigen (CEA), neoadjuvant chemotherapy, adjuvant chemotherapy, postoperative complications, and inflammatory score. Patients who achieved R0 resection of liver metastasis received 5-fluorouracil-based adjuvant chemotherapy within 2 months of the initial surgery, regardless of the presence or absence of preoperative chemotherapy. The type of adjuvant chemotherapy regimen (5-FU monotherapy or 5-FU plus oxaliplatin) depended on the patient’s performance status, patient’s choice, and the out-patient doctor’s decision. Synchronous (as opposed to metachronous) CRLM was defined as simultaneous presentation of liver metastases at the time of CRC surgery. Location was classified as colon (cecum to rectosigmoid colon) or rectum. Baseline laboratory data, including neutrophils, lymphocytes, LDH, albumin, and CRP were also compared between the groups.

Statistical analysis was performed using Bell Curve for Excel software, version 2.02 (Social Survey Research Information Co., Ltd., Tokyo, Japan). Data are presented as the median value and range. Differences in categorical variables were compared using Fisher’s exact test or the chi-squared test, as appropriate. Differences in continuous variables were analyzed with the Mann–Whitney *U*-test. PFS was defined as the time from R0 resection of CRLM to the appearance of new recurrent metastases or death from any cause. OS was defined as the time from surgery to death or last follow-up visit. RFS and OS were calculated according to the Kaplan–Meier method. Differences between groups were tested for significance using the log-rank test. Multivariate analysis using a Cox hazards model was used to identify independent prognostic markers for patients with CRLM. Clinical variables with a *p* value <0.20 in univariate analysis were included in the multivariate analysis. All *p* values <0.05 were considered significant.

## Results

**Fig 1:**
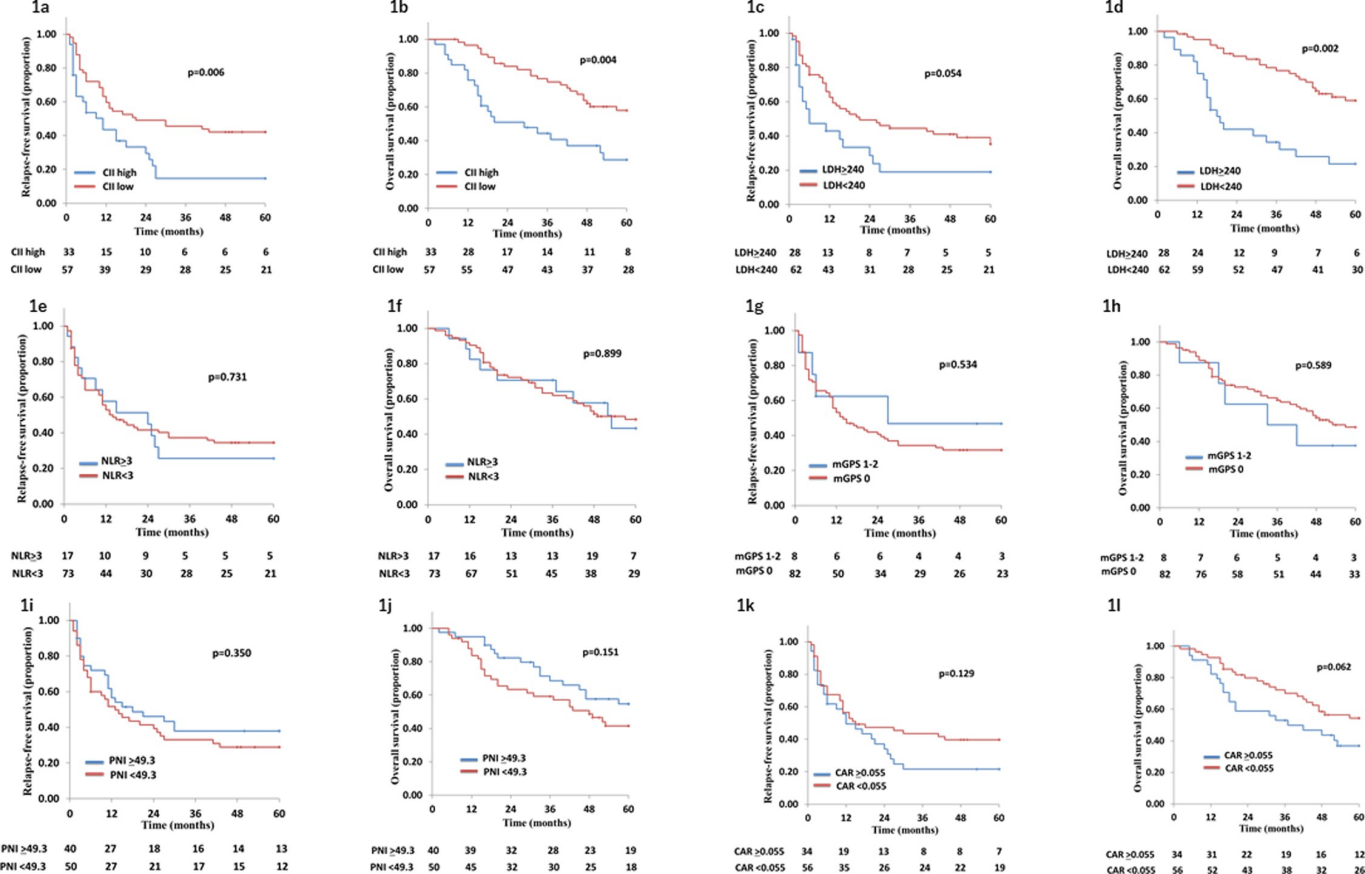
Survival curves for 5-year relapse-free survival (a: CII, c: LDH, e: NLR, g: mGPS, i: PNI, and k: CAR) and for 5-year overall survival (b: CII, d: LDH, f: NLR, h: mGPS, j: PNI, and l: CAR). CII; colon inflammatory index, NLR; neutrophil-to-lymphocyte ratio, mGPS; modified Glasgow prognostic index, PNI; prognostic nutritional index, CAR; CRP-to-albumin ratio.

The median follow-up period was 44 months (2–101 months). Fig 1 shows survival curves for each IBS subgroup. Five-year-RFS (CII2; 12.5%, CII1; 14.5%, CII0; 42.9%) and OS (CII2; 32.4%, CII1; 25.4%, CII0; 57.7%) were significantly shorter in the high CII group than in the low CII group (p = 0.021 and p = 0.006, respectively). Five-year-OS was significantly shorter in patients with high LDH than in those with low LDH (20.0% vs 54.4%, p = 0.002); however, 5-year-RFS was similar between the groups (22.6% vs 32.7%, p = 0.064). Regarding the other types of IBS, there were no significant differences in 5-year-RFS and 5-year-OS between high (-H) and low (-L) score groups, or in NLR (5-year-RFS: NLR-H 25.6% vs NLR-L 33.5%, p = 0.761; 5-year-OS: NLR-H 40.7% vs NLR-L 47.3%, p = 0.926), mGPS (5-year-RFS: mGPS-H 30.8% vs mGPS-L 46.8%, p = 0.508; 5-year-OS: mGPS-H 47.3% vs mGPS-L 28.5%, p = 0.368), PNI (5-year-RFS: PNI-H 36.3% vs PNI-L 28.6%, p = 0.418; 5-year-OS: PNI-H 53.0% vs PNI-L 40.9%, p = 0.189), and CAR (5-year-RFS: CAR-H 21.6% vs CAR-L 38.6%, p = 0.149; 5-year-OS: CAR-H 35.9% vs CAR-L 53.2%, p = 0.080).

Table 1 shows a comparison of clinical characteristics between the CII high and CII low groups. CEA level was significantly higher in the CII high group than the CII low group (12.4 vs 7.3, p = 0.004). Laboratory data including neutrophils (3280 vs 2440, p = 0.005), LDH (258 vs 192, p<0.001), and CRP (0.27 vs 0.07, p = 0.009) were significantly higher and lymphocytes (1230 vs 1510, p<0.001) were significantly lower in the CII high group. Other factors including sex, age, location of primary tumor, pathological T/N status, type of liver metastasis, surgical procedure, number of liver metastases, perioperative chemotherapy, and postoperative complications were similar between the groups.

**Table 1:** Clinicopathological characteristics according to colon inflammatory index.

	High CII (n = 33)	Low CII (n = 57)	*p* value
Sex			0.363
Male	19 (57.6)	39 (68.4)	
Female	14 (42.4)	18 (31.6)	
Age (y)	65 (31–87)	65 (32–82)	0.654
Location of primary tumor			0.263
Colon	23 (69.7)	32 (56.1)	
Rectum	10 (30.3)	25 (43.9)	
Pathological T status			0.897
pT1–3	24 (72.7)	40 (70.2)	
pT4	7 (21.2)	12 (21.1)	
Unknown	2 (6.1)	5 (8.8)	
Pathological N status			0.938
Negative	11 (33.3)	17 (29.8)	
Positive	21 (63.6)	38 (66.7)	
Unknown	1 (3.0)	2 (3.5)	
Type of liver metastasis			0.659
Synchronous	21 (63.6)	33 (57.9)	
Metachronous	12 (36.4)	24 (42.1)	
Surgical procedure			0.295
Lobectomy	9 (27.3)	10 (17.5)	
Partial resection	24 (72.7)	47 (82.5)	
Liver metastases	1 (1–7)	1 (1–6)	0.491
CEA (ng/mL)	12.4 (1.7–176.5)	7.3 (1.5–63.5)	0.004
Neoadjuvant chemotherapy			0.514
No	21 (63.6)	41 (71.9)	
Yes	12 (36.4)	16 (28.1)	
FU monotherapy	1 (0.03)	2 (0.04)	
FU plus oxaliplatin	11 (33.3)	14 (24.6)	
Anti-VEGF antibody	5 (15.1)	9 (15.8)	
Adjuvant chemotherapy			
No	25 (75.8)	30 (52.6)	
Yes	8 (24.2)	27 (47.3)	
FU monotherapy	2 (0.06)	14 (24.6)	
FU plus oxaliplatin	6 (0.2)	13 (22.8)	
Postoperative complications			1.000
No	27 (81.8)	47 (82.5)	
Yes	6 (18.2)	10 (17.5)	
Laboratory data			
LDH	258 (112–753)	192 (78–285)	<0.001
Albumin	4.0 (3.3–5.2)	4.1 (3.3–5.1)	0.514
C-reactive protein	0.27 (0.02–3.31)	0.07 (0.01–1.50)	0.009
Inflammatory score			
NLR	2.92 (0.93–5.46)	1.71 (0.58–3.55)	<0.001
PNI	46.7 (36.4–59.2)	49.4 (38.9–61.1)	0.026
CAR	0.067 (0.004–0.923)	0.017 (0.002–0.424)	0.009
mGPS (0/1/2)	28 (84.8)/3 (9.1)/2 (6.1)	54 (94.7)/2 (3.5)/1 (1.8)	0.278

Data are presented as the number (%) or the median (range). CII; colon inflammatory index, CEA; carcinoembryonic antigen, LDH; lactate dehydrogenase, NLR; neutrophil-to-lymphocyte ratio, PNI; prognostic nutritional index, CAR; CRP-to-albumin ratio, mGPS; modified Glasgow prognostic index

Table 2 shows the results of univariate and multivariate analyses for predicting RFS. CII score (p = 0.008) was significantly associated with RFS in univariate analysis. Multivariate analysis also showed that CII score (hazard ratio 2.128, 95%CI 1.147–3.430, p = 0.015) was an independent predictor of RFS.

**Table 2:** Univariate and multivariate analysis for predicting relapse-free survival.

	Univariate analysis			Multivariate analysis		
	Hazard ratio	95% CI	*p* value	Hazard ratio	95% CI	*p* value
Age ≥70 y	0.959	0.435–2.114	0.919			
Sex (female vs male)	1.026	0.601–1.748	0.924			
Location of primary tumor (colon vs rectum)	1.157	0.688–1.974	0.580			
Pathological T status (T1–3 vs T4)	1.557	0.865–2.803	0.139	1.700	0.903–3.202	0.099
Pathological N status (negative vs positive)	1.306	0.750–2.274	0.344			
Type of liver metastasis (synchronous vs metachronous)	0.698	0.411–1.185	0.183	0.722	0.424–1.227	0.229
Postoperative complications (no vs yes)	0.738	0.295–1.848	0.517			
Perioperative chemotherapy (no vs yes)	0.828	0.496–1.382	0.470			
CEA ≥5 ng/ml	0.990	0.580–1.687	0.971			
Surgical procedure (partial resection vs lobectomy)	1.075	0.580–1.991	0.817			
mGPS (0 vs 1–2)	0.728	0.353–1.499	0.389			
PNI (low vs high)	0.807	0.479–1.357	0.419			
CAR (low vs high)	1.459	0.871–2.444	0.151	0.962	0.525–1.763	0.905
CII (0 vs 1–2)	2.007	1.192–3.376	0.008	2.128	1.147–3.430	0.015

CI; confidence interval, CEA; carcinoembryonic antigen, mGPS; modified Glasgow prognostic index, PNI; prognostic nutritional index, CAR; CRP-to-albumin ratio, CII; colon inflammatory index

Table 3 shows the results of univariate and multivariate analyses for predicting OS. CII score (p = 0.005) was significantly associated with RFS in univariate analysis. Multivariate analysis also showed that CII score (hazard ratio 2.639, 95%CI 1.331–5.323, p = 0.005) was an independent predictor of OS.

**Table 3:** Univariate and multivariate analysis predicting overall survival.

	Univariate analysis				Multivariate analysis		
	Hazard ratio	95% CI	*p* value	Hazard ratio		95% CI	*p* value
Age ≥70 years	1.311	0.554–3.104	0.536				
Sex (female vs male)	1.219	0.670–2.217	0.515				
Location of primary tumor (colon vs rectum)	1.353	0.776–2.358	0.286				
Pathological T status (T1–3 vs T4)	1.628	0.872–3.040	0.125	1.800		0.893–3.628	0.100
Pathological N status (negative vs positive)	0.870	0.482–1.571	0.644				
Type of liver metastasis (synchronous vs metachronous)	0.850	0.482–1.498	0.575				
Postoperative complication (no vs yes)	1.385	0.589–3.256	0.455				
Perioperative chemotherapy (no vs yes)	0.664	0.381–1.160	0.151	0.596		0.325–1.091	0.093
CEA ≥5 ng/ml	1.151	0.639–2.073	0.639				
Surgical procedure (partial resection vs lobectomy)	1.365	0.707–2.633	0.352				
mGPS (0 vs 1–2)	1.011	0.524–1.951	0.971				
PNI (low vs high)	0.680	0.384–1.205	0.186	1.068		0.566–2.015	0.878
CAR (low vs high)	1.644	0.941–2.872	0.080	1.126		0.584–2.173	0.722
CII (0 vs 1–2)	2.211	1.259–3.883	0.005	2.639		1.331–5.323	0.005

CI; confidence interval, CEA; carcinoembryonic antigen, mGPS; modified Glasgow prognostic index, PNI; prognostic nutritional index, CAR; CRP-to-albumin ratio, CII; colon inflammatory index

## Discussion

In the present study, we examined the correlation between prognosis after R0 resection for CRLM and IBS including CII, mGPS, PNI, and CAR. Survival curves and the log-rank test revealed a correlation of high CII level with poor prognosis. Multivariate analysis of RFS and OS revealed CII score as an independent prognostic factor.

CII is a novel IBS that has been reported to predict prognosis and treatment efficacy in patients with CRC [9]. Casadei-Gardini et al. examined 276 patients with metastatic CRC and investigated the correlation between CII score and prognosis [9]. Median PFS was 10.3 months, 8.7 months, and 7.3 months in patients with CII0 (good), CII1 (immediate), and CII2 (poor), respectively (p<0.001). Median OS was 29.9 months, 20.9 months, and 14.4 months in patients with CII0, CII1, and CII2, respectively (p<0.001). Multivariate analysis revealed CII as an independent prognostic factor for predicting PFS and OS. They concluded that CII score was a good prognostic marker for metastatic CRC patients [9]. In agreement with those results, the present study of patients who underwent R0 resection for CRLM also found that high CII score was an independent prognostic factor.

CII consists of two components, NLR and LDH. NLR is a simple IBS that is calculated using neutrophil count and lymphocyte count [12]. The patient’s immune system is an important factor that correlates with cancer prognosis [13, 14]. Neutrophilia usually occurs during systemic inflammation, and lymphopenia is an important marker of depressed cell-mediated immunity [15]. High lymphocyte count is reported to be associated with good prognosis, whereas low lymphocyte count is associated with poor prognosis [16]. In addition, neutrophils suppress lymphocyte-mediated cytolysis, which has a reported association with poor prognosis [17]. The role of NLR in cancer prognosis due to immune escape of tumor from tumor-infiltrating lymphocytes, lymphocyte count related to disease severity, and tumor angiogenesis activity of tumor-induced neutrophils [17]. Several reports have examined the correlation between NLR and prognosis in CRC patients [12, 18]. Mazaki and colleagues examined the prognostic value of NLR in 375 CRC patients with Stage II–III disease [19]. Five-year-RFS and 5-year-OS were longer in the low-NLR group than the high-NLR group (RFS: 87.8% vs 77.9%, p = 0.032; OS: 94.5% vs 87.0%, p = 0.042). In the present study, NLR was not correlated with prognosis. One possible explanation for this finding is the different backgrounds of the CRLM patients who underwent R0 resection, including variation in type of metastasis (synchronous or metachronous), number of liver metastases, liver procedure (lobectomy or partial resection), presence or absence of perioperative chemotherapy, and regimen of chemotherapy (5FU monotherapy, 5FU plus oxaliplatin, the use of anti-VEGF antibody). For this reason, a combined IBS such as CII might show better performance than a simple scoring system for predicting outcomes in the situation of heterogeneity in complex patient cohorts.

LDH is the other factor in CII. LDH is a cytoplasmic enzyme that is involved in tumor metabolism and initiation [20]. Serum LDH level is indirectly associated with neo-angiogenesis, tumor hypoxia, metastasis development, and poor prognosis in several cancers [20]. In colorectal cancer, a high LDH value was shown to indicate a hypoxic microenvironment that resulted in poor prognosis for metastatic CRC treated with chemotherapy [10]. However, this was not evident in patients treated with chemotherapy plus anti-VEGF antibody, suggesting that LDH might be a potential predictor of benefit from VEGF signaling inhibitor [20]. Our results showed similar 5-year-RFS between the LDH-L and LDH-H groups; however, OS was worse in the LHD-H group than the LDH-L group. This result might have been due to treatment bias, including variation in the use of anti-VEGF antibody both perioperatively and after recurrence.

We also examined the correlation between IBS using CRP and/or albumin including PNI, CAR, mGPS and cancer prognosis. CRP is an acute phase protein synthesized in the liver that can stimulate inflammatory cytokines and lead to tumor deterioration [21]. In contrast, albumin is a major indicator of nutritional status that is related to the systemic inflammatory response [22]. Hypoalbuminemia usually reflects insufficient oral intake and excessive tumor consumption that induces inflammatory cytokines such as IL-1, IL-6, and TNF-α [23]. A previous study found that CRP/albumin-based IBS such as PNI, CAR, and mGPS were closely correlated with prognosis in CRC patients [8, 24, 25]. However, in our study, these scores were not correlated with prognosis. Our cohort included selected patients with CRLM who had undergone R0 resection, and excluded inoperable patients. Before liver surgery, we improved the patient’s nutritional status and general condition to enable them to better tolerate invasive surgery. Indeed, the serum CRP/albumin level was normal in almost all of the present patients, with median CRP status of 0.1 (range, 0–3.3) and albumin status of 4.1 (range, 3.3–5.2), which might have influenced our results.

Perioperative chemotherapy has been reported for CRLM [26–28]. The EORCT study revealed a 9.2% increase in the 3-year-RFS rate, from 33.2% with surgery alone to 42.4% with surgery plus perioperative chemotherapy using FOLFOX, in patients with CRLM who underwent liver resection [26]. However, later reports showed that 5-year OS was 51.2% in the preoperative chemotherapy group compared with 47.8% in the surgery alone group, and the difference was not significant [29].

Three recent RCTs examined oncological outcomes between patients in a surgery-alone group and a group that had adjuvant chemotherapy after R0 resection for CRLM [26–28]. All three studies reported longer RFS in the adjuvant chemotherapy group; however, OS was similar among the groups [26–28]. In our study, perioperative chemotherapy was not a prognostic factor for RFS or OS, possibly for the reason that only 55.5% of patients with CRLM received perioperative chemotherapy, which suggests several selection biases. In addition, the duration of chemotherapy and regimen type varied according to the physician’s discretion and patient’s wishes. The present study also showed that patients with CII-H had high risk of poor prognosis. Therefore, the choice of appropriate regimen and duration of perioperative chemotherapy could be crucial for improving the prognosis of CII-H patients.

There are several limitations of the present study. First, it was retrospective study and the sample size was small. It is necessary to conduct a multicenter study with a large sample size in the future. Second, the decision of whether to undergo perioperative chemotherapy and selection of the chemo-regimen was at the discretion of the surgeon, without clear criteria. Third, the original CII score was compared by dividing the patients into three groups: score 0 (good), 1 (intermediate), and 2 (poor). In the present analysis, CII0 was defined as "low risk" and CII1–2 as "high risk" because of the small number of patients with CII2 and the inability to generate adequate statistics. Further studies are required to clarify these issues.

## Conclusion

In conclusion, CII could be used as a prognostic marker in CRLM patients with R0 liver resection. Further studies are needed to evaluate appropriate indications for surgery and perioperative treatment.

## Supporting information

S1 Data (XLSX)

S1 Table Correlations between preoperative CEA level and markers of systemic inflammation. (DOCX)
