# Supplementary material for: Colon inflammatory index as a useful prognostic marker after R0 resection in patients with colorectal cancer liver metastasis
Source: PLoS One. 2022 Oct 4;17(10):e0273167. doi: 10.1371/journal.pone.0273167 (PMC9531835; doi:10.1371/journal.pone.0273167)
Supplement: S1 Table — (DOCX) [file pone.0273167.s002.docx]

Supplementary Table 1. Correlations between preoperative CEA level and markers of systemic inflammation

|  | Preoperative CEA level | |  |
| --- | --- | --- | --- |
| Marker | Pearson’s r | p value |  |
| Serum albumin | –0.123 | 0.251 |  |
| Serum C-reactive protein | 0.559 | <0.001 |  |
| Blood lymphocyte count | –0.144 | 0.178 |  |
| Serum lactate dehydrogenase | 0.240 | 0.053 |  |
| PNI | –0.173 | 0.107 |  |
| CAR | 0.539 | <0.001 |  |
| mGPS | 0.568 | <0.001 |  |
| NLR | 0.188 | 0.078 |  |
| CII | 0.388 | <0.001 |  |

CEA, carcinoembryonic antigen; PNI, Prognostic nutritional index; mGPS, modified Glasgow prognostic index; NLR, Neutrophil to lymphocyte ratio; CII, colon inflammatory index
